# Female urethral stricture: A multi‐centre experience and lessons learnt

**DOI:** 10.1002/bco2.70024

**Published:** 2025-04-29

**Authors:** Madeleine Bain, Daniel Esteban Gomez Zapata, Kapilan Ravichandran, Cora Fogaing, Apurva Anand, Amey Talpallikar, Shreyas Bhadranawar, Sanjay Kulkarni, Devang Desai, Pankaj Joshi

**Affiliations:** ^1^ Toowoomba Hospital Queensland Australia; ^2^ Kulkarni Reconstructive Urology Center Pune India; ^3^ University of Queensland Queensland Australia; ^4^ Griffith University Queensland Australia; ^5^ University of Southern Queensland Queensland Australia

**Keywords:** buccal mucosa, female urethroplasty, graft, reconstructive urology, urethra, urethral stricture

## Abstract

**Objectives:**

To review demographics, surgical techniques and outcomes of female patients undergoing buccal mucosal graft substitution urethroplasty.

**Materials and methods:**

An international multi‐institutional study was performed through a retrospective review of a prospectively managed database of female urethroplasty outcomes at two sites from December 2016 to June 2023. Institutions included a high‐volume tertiary referral centre performing 500 urethroplasties annually, and a regional centre with a fellowship‐trained urethroplasty surgeon performing ~50 urethroplasties annually. Female urethroplasty accounted for 2% of urethroplasties performed, utilising dorsal onlay, ventral inlay and double‐face techniques.

**Results:**

Forty‐two patients underwent female urethroplasty between 2016 and 2023; 20 dorsal onlay grafts, 14 ventral inlay grafts and 8 double‐face urethroplasty. The mean age was 45 years (SD 12.07) and mean follow‐up 27 months (SD 17.22). The most common aetiology was idiopathic in 59%. The most common presenting symptom was obstructive lower urinary tract symptoms in 86%. Urethral dilatations were the most common treatment before urethroplasty, with a mean of 9 (SD 1.2) dilations pre‐urethroplasty. Stricture locations seen were; proximal 7%, proximal to mid‐14%, mid‐31%, mid to distal 10% and distal 38%. A total of 88% were successful overall; dorsal onlay was 100%, ventral inlay urethroplasties 71% and double‐face 88%. Mean Qmax improvement was 291% at 6 months. In those who required dilatations or further surgery postoperatively (n = 5); four were ventral inlay (one mid‐distal, three distal), and one double‐face distal stricture. All patients including those requiring secondary treatments were continent and did not require intermittent self‐catheterisation or suprapubic catheter insertion.

**Conclusion:**

Urethroplasty is an effective long‐term therapeutic option for managing female urethral strictures. Dorsal onlay urethroplasty demonstrated the highest success rate, and stands out as a versatile technique, addressing distal to proximal urethral strictures. However, the chosen urethroplasty technique should be tailored to the characteristics of the stricture, patient and surgeons experience.

## 1 INTRODUCTION

Bladder outlet obstruction is a relatively rare condition among women with lower urinary tract symptoms, comprising approximately 2.7%–8% of cases, with female urethral stricture disease contributing to approximately 18%–40%.^1^ Considering its rarity, there lack of standardised definition, diagnostic criteria or treatment protocols. It has been described as a fixed anatomical narrowing between the bladder neck and distal urethra. In clinical practice, various definitions have been employed, encompassing singularly or in combination: symptomatic presentation, a maximum flow rate of less than 12 ml/sec, inability to calibrate the urethra at 14 Fr, cystoscopic evidence of stricture, or the observation of proximal dilation with stricture during voiding cystourethrogram (VCU).^2^, ^3^, ^4^

Traditionally, the management of these patients has revolved around dilations and endoscopic procedures, which have demonstrated limited success rates. The success rate of initial urethral dilatation stands at 41%, with decreasing success rates to 14.9% noted in cases with a history of multiple prior dilatations.^4^ Additionally, these patients often necessitate recurrent dilations, with an average interval of 15 months between interventions.^5^ Repeated surgical interventions, bothersome symptoms, complications of bladder outlet obstruction and impact on sexual function can significantly impact the quality of life for patients with urethral strictures. Consequently, there has been a growing interest in the utilisation of reconstructive techniques, involving the application of flaps, vaginal grafts or buccal grafts, which have reported success rates exceeding 80%.^4^, ^6^

In this study, we aim to present a series of 42 female patients with urethral stricture disease treated with urethroplasty and buccal mucosal graft. Our analysis encompasses the evaluation of success rates, identification of potential complications and the exploration of the advantages conferred by different surgical approaches.

## 2 MATERIALS AND METHODS

**FIGURE 1:**
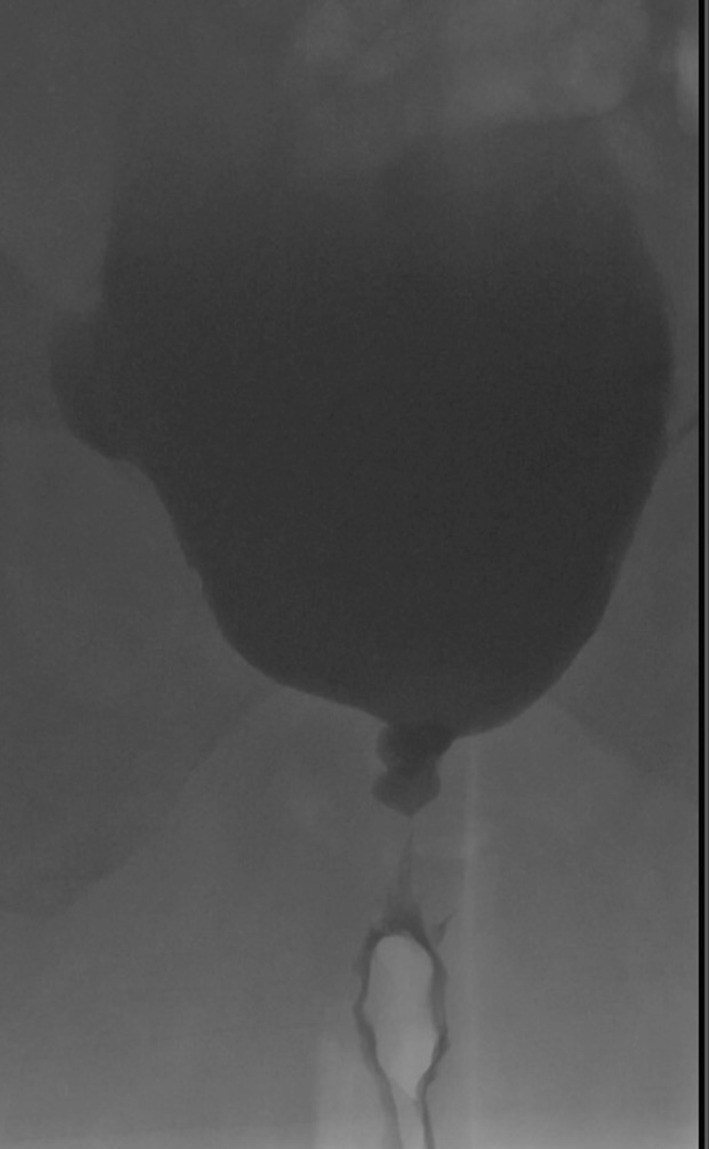
Wine glass image. Voiding cystourethrography with a distal female urethral stricture and prestenotic dilation.

A cohort of 42 female patients who underwent urethral reconstruction at two centres from December 2016 to June 2023 were retrospectively analysed from a prospectively identified and maintained dataset. Before surgical intervention, all patients underwent a comprehensive pre‐operative assessment, which encompassed physical examination, uroflowmetry, voiding cystourethrogram and urethroscopy to identify the location, calibre and possible aetiology of the stricture. Inclusion criteria comprised patients symptomatic with urethral stricture disease and exhibiting a maximum flow rate (Qmax) of less than or equal to 12 ml/second. Stricture must have been confirmed by urethroscopy, which also provided information on stricture location and characteristics. Subsequently, a voiding cystourethrogram (VCU) was performed to further elucidate the precise location and calibre of the stricture. Figure 1 presents a “wine glass image” of a voiding cystourethrogram, with proximal pre‐stenotic dilatation to the female urethral stricture. Based on these findings, the surgical approach was planned, with the selection of one of the three following techniques: dorsal wall onlay, ventral inlay or double‐face urethroplasty.

Postoperatively, all patients had their catheters removed at 4 weeks and received prophylactic antibiotic therapy. On the day of catheter removal and subsequently, every 3 months, uroflowmetry and post‐void residual volume measurements were undertaken. A successful procedure was defined by a combination of; symptomatic improvement, uroflowmetry results exceeding the maximum flow rate (Qmax) of 15 ml/second and the absence of any need for additional interventions. Data analysis was performed, presenting categorical variables as frequencies and percentages, while continuous variables were reported as mean values accompanied by standard deviation. Fisher's exact test was used to compare overall success rates. Ethical approval was obtained from the relevant institutional review boards for both centres – HREC: LNR/2023/QTDD/102790.

## 3 SURGICAL TECHNIQUE

The choice of surgical approach was guided by careful consideration of the specific characteristics and location of the urethral strictures (Table 1). In instances where strictures were identified in the proximal urethra involving the bladder neck, it was advisable to avoid urethroplasty due to the elevated risk of incontinence. For proximal strictures not involving or very close to the bladder neck, a dorsal onlay technique was utilised. For dorsal strictures spanning the mid to distal regions of the urethra, the preferred technique was the dorsal onlay urethroplasty. In cases where strictures were identified ventrally within the mid to distal segments, a ventral onlay urethroplasty was the recommended approach. Distal strictures near obliterative or obliterative were managed through the utilisation of the double‐face urethroplasty technique. The selection of the most suitable surgical technique was made with the primary objective of optimising treatment outcomes, taking into account the specific anatomical and pathological attributes of the stricture site.

**TABLE 1:** Patient and stricture characteristics.

Patient characteristics (N = 42)	
Age (mean)	45 (SD ‐ 12.7)

Aetiology	Number (%)
Idiopathic	25 (59)
Iatrogenic	13 (31)
Traumatic	2 (5)
Lichen sclerosis	2 (5)

Symptoms (%)	Number (%)
Lower obstructive Symptoms	36 (86)
Urinary infection	16 (38)
Urinary retention	4 (10)
Urinary incontinence	2 (5)

Previous treatment	Number (%)
Dilations	28 (67)
Direct vision internal urethrotomy	2 (5)
Urethroplasty	3 (7)

Comorbidities	Number (%)
Diabetes	5 (12)
Hypertension	11 (26)

Location of the stricture	Number (%)
Proximal	3 (7%)
Proximal‐mid	6 (14%)
Mid	13 (31%)
Mid‐distal	4 (10%)
Distal	16 (38%)

In all cases, the procedures were conducted under general anaesthesia. Regional anaesthesia was applied at the site of the buccal mucosal graft and the operative site. Patients were positioned in the lithotomy position. All operating surgeons underwent fellowship training in the surgical techniques employed.

### 3.1 Dorsal Onlay

**FIGURE 2:**
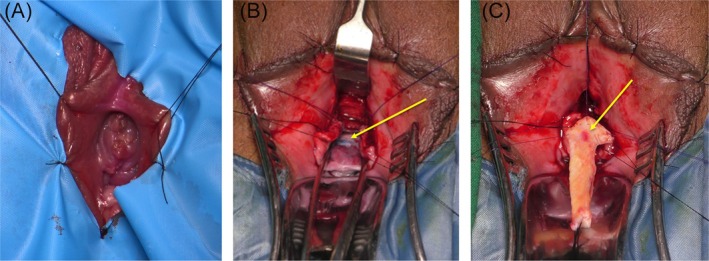
Dorsal onlay urethroplasty technique. (A) Stay sutures are placed to retract the vaginal labia to aid exposure of the urethral opening, vestibule and introitus. (B) Dorsal urethral wall has been incised (marked by yellow arrow). Dissection has been carried out above the urethra. (C) Buccal mucosal graft (marked by yellow arrow) which has been tailored to the urethrotomy size, and incorporated dorsally, initially proximally and then to the urethral edges bilaterally.

The initial step involved the insertion of a guidewire into the urethra to confirm the presence of the stricture. Subsequently, endoscopy was performed using a 4.5 to 7 Fr scope to assess the precise location, length and characteristics of the stricture. If the calibre of the stricture did not permit a scope, the guidewire was passed then the urethra dissected and stricture incised. For improved exposure, as depicted in Figure 2A, stay sutures were strategically placed to retract the vaginal labia. To aid in stricture identification, a combination of suprameatal lidocaine with epinephrine and methylene blue injection was employed. An inverted U‐shaped incision was made above the meatus, followed by dissection just above the urethra and beneath the corporal bodies of the clitoris. The dorsal onlay technique commenced with the exposure of the dorsal urethral wall and an incision made at the 12 o'clock position across the stricture, as illustrated in Figure 2B. The length of the urethrotomy was carefully measured, and an individualised inner buccal graft was harvested from the buccal mucosa of the patient's cheek. This graft was subsequently inserted into the proximal edge of the urethrotomy and secured in place, ensuring proper apposition to the urethral mucosa on both sides as evidenced in Figure 2C. Quilting was achieved through the use of multiple separate Vicryl stitches across the graft and corporal bodies to ensure robust fixation. The meatus was approximated to the borders of the incision. A 16 Fr indwelling catheter is placed.

### 3.2 Ventral inlay

**FIGURE 3:**
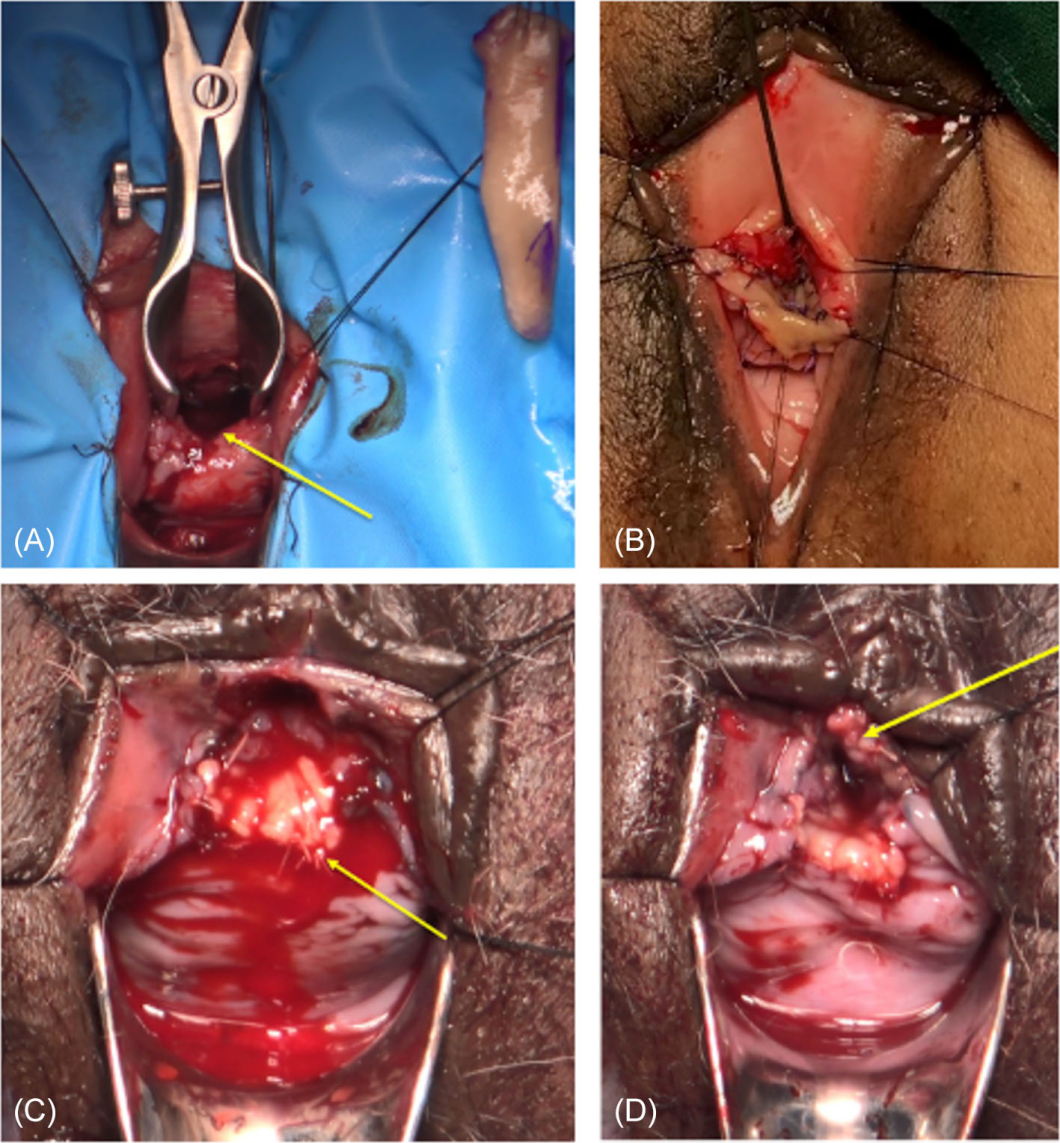
Ventral inlay and double‐face urethroplasty techniques. (A) Ventral inlay ‐ ventral urethra has been incised and urethrotomy site marked by yellow arrow, buccal mucosal graft prepared for parachuting into urethrotomy site. (B) Ventral inlay ‐ buccal mucosal graft incorporated with inlay technique to ventral urethra. Sutures placed to lateral borders of urethrotomy and metal edge anteriorly. (C) Double‐face ‐ ventral inlay component of double‐face technique, (incorporated buccal mucosal graft marked in yellow). (D) Double‐face ‐ dorsal inlay incorporation of buccal mucosal graft completed (incorporated dorsal graft marked in yellow).

The ventral inlay approach involves the initial placement of stay sutures along the meatal border to ensure adequate exposure. Subsequently, the ventral urethral mucosa is incised from the urethral lumen at the 6 o'clock position, extending across the stricture until the passage of a 24 Fr dilator becomes unobstructed, as visually demonstrated in Figure 3A. Following this step, Vicryl sutures are meticulously positioned at the 11, 12 and 1 o'clock positions along the proximal border of the urethrotomy, and the buccal graft is introduced into the defect. The graft's edges are precisely approximated to the lateral borders of the urethrotomy, as exemplified in Figure 3B. Finally, a 16 Fr indwelling urethral catheter is carefully inserted.

### 3.3 Double‐face

The double‐face buccal graft urethroplasty, initially delineated in 2020 by Joshi and Kulkarni, represents a surgical procedure that combines elements of both dorsal inlay and ventral inlay techniques.^7^ The approach commences with a dorsal incision of the urethra, specifically at the 12 o'clock position, extending across the stricture to gain access to the ventral urethral wall. Subsequently, an incision is made in the ventral mucosa, followed by the fixation of the initial graft using an inlay technique (Figure 3C). This is then followed by the completion of the dorsal wall using an inlay graft (Figure 3D).

## 4 RESULTS

Clinical records of 42 patients between 2016 and 2023 who underwent female urethroplasty were analysed. Mean age of patients was 45.35 years old (SD ‐ 12.07) and mean follow‐up was 27 months (SD‐17.22). Aetiology of stricture was mainly identified as idiopathic in 59%, iatrogenic in 31%, traumatic in 5% and lichen sclerosis in 5% (Table 1).

The majority of patients included in the study had received prior intervention for their stricture. A total of 28 patients (67%) had undergone dilations. The mean number of dilatations is 9.0 (SD‐ 1.2) before urethroplasty. Three patients (7%) had previous urethroplasty, and two patients (5%) had undergone previous direct visual internal urethrotomy (DVIU). Among the patients, five individuals (12%) had a history of diabetes mellitus as a premorbid medical condition.

In the study cohort, the primary site of stricture was the distal urethra in 16 patients (38%), followed by mid urethra in 13 patients (31%) then proximal to mid urethra in 6 patients (14%), mid‐to‐distal urethra in 4 patients (10%) and proximal urethra in 3 patients (7%). All surgical procedures involved the utilisation of buccal mucosal grafts, with 20 patients (48%) undergoing dorsal onlay graft urethroplasty, 14 patients (33%) undergoing ventral inlay urethroplasty and 8 patients (19%) undergoing double‐face urethroplasty. The techniques utilised for each location are as follows; distal (6 ventral inlay, 7 double‐face and 1 dorsal onlay), mid (13 dorsal onlay), proximal (3 dorsal onlay), mid to distal (1 double‐face, 3 dorsal onlay) and mid to proximal (6 dorsal onlay).

The overall success rate, defined as the absence of the need for dilations or additional procedures post‐surgery to ensure that the patient is no longer symptomatic of obstructive lower urinary tract symptoms or Qmax >12 mls/second; is 88% across all operative techniques. In subgroup analysis, the success rate was 100% for dorsal onlay urethroplasty (95% confidence interval 0.84–1.0), 88% for double‐face urethroplasty (CI 0.53–0.98), and 71% for ventral inlay (CI 0.45–0.88) (Table 2). There was a statistically significant difference in successful outcomes for dorsal onlay assessed with the Fishers Exact test (p = 0.022). Wide 95% confidence intervals were reported, likely due to the limited sample size. The techniques and locations of those patients (n = 5) who were not successful (requiring dilatations and additional procedures post‐surgery); four ventral inlays which were one mid‐distal and three distal, one double‐face which was distal in location. Three out of the five patients required one further urethral dilatation post‐operatively to ensure that they were able to void well post‐operatively. Two out of the five which underwent ventral inlay both in distal locations required a secondary urethroplasty (one double‐face and one dorsal), which were both successful.

**TABLE 2:** Results.

	Number (%)
Total surgeries	42 (100)
Dorsal onlay	20 (48)
Ventral inlay	14 (33)
Double‐face (Dorsal onlay + ventral inlay)	8 (19)
All surgeries success (%)	37/42 (88)
Dorsal onlay	20/20 (100%)
Ventral Inlay	10/14 (71%)
Double‐face	7/8 (88%)

Uroflow	Mean (SD)
Pre‐surgery uroflow Qmax	9.1 (2.9)
Post‐surgery uroflow Qmax	24.5 (7.7)
Post‐void Residual volume	Mean (SD)
Pre‐surgery	110 (15)
Post‐surgery	15 (8)

No intra‐operative complications were identified, and procedures had an average duration of 50 minutes. The extent of blood loss was minimal (<50 ml), obviating any need for transfusion or a return to the operating room. Patients were typically discharged within the first or second day following the surgical intervention and no major complications were observed in the postoperative period. One uncomplicated urinary tract infection, two minor bleeding from the graft donor site and one partial dehiscence of the wound were all managed conservatively with full resolution of symptoms.

No patients at the end of treatment were incontinent, required clean intermittent self‐catheterisation or a suprapubic catheter insertion. Uroflowmetry results showed an average increase from 9.1 ml/sec before surgery to 26.5 ml/sec post‐surgery at 3 months which demonstrates a 291% improvement. The average post‐void residual volume decreased from 110 ml (S.D 15) to 15 ml (S.D 8).

## 5 DISCUSSION

In recent years, female urethral stricture has garnered increasing attention as an established aetiology of lower urinary tract symptoms. While dilation and endoscopic treatments have been the conventional choices due to their familiarity and relative simplicity, the emergence of reconstructive techniques boasting high success rates underscores the significance of considering urethroplasty as a treatment option.^1^ The success rates of urethroplasty in the treatment of female urethral stricture have been reported to range from 62% to 100% in the existing literature. However, the definition of success varies among studies and is generally described as the resolution of symptoms without the need for further interventions, along with a uroflow rate exceeding 15 ml/sec.^3^, ^4^, ^5^, ^6^, ^7^, ^8^, ^9^, ^10^ In this study, which had a mean follow‐up duration of 27 months, the overall success rate, defined as the absence of dilation or additional procedures (and Qmax >15 mls/sec), was 88% for all surgical techniques. At the time of writing, all patients are still engaging with follow‐up since their procedure. In a subgroup analysis, the success rates were 100% for dorsal onlay urethroplasty and 88% for double‐face and 71% for ventral inlay urethroplasties (Table 2). In unsuccessful procedures, the mean time to failure of 12.8 months.

There are several advantages to consider when performing dorsal female urethroplasty. It allows surgical access to the entire length of the urethra, preserving the ventral urethral wall for potential future incontinence procedures,^11^, ^12^, ^13^, ^14^ eliminates the risk of urethrovaginal fistula,^9^, ^14^ and provides good graft support and blood supply from the flat surface of the clitoris.^14^ Its versatility affords it as an option for most strictures. Opposed to the ventral approach, the incision is not just mucosal‐based and therefore can be utilised for strictures with a narrower calibre. Other series of dorsal urethroplasty approaches have reported successful outcomes in greater than 80% of patients, with 86% success at an average 21 months of follow‐up reported by Gomez and 83% over 12 months by Higgins.^15^, ^16^

Some authors argue in favour of ventral approaches to avoid potential damage to the clitoral cavernosal nerves during dorsal dissection, which could lead to a decrease in sexual function.^10^ However, T. Manasa et al. reported an improvement in sexual function means following dorsal onlay urethroplasty, indicating improved sexual function.^17^ Similarly, Mittal A. et al. reported similar results with no patients experiencing sexual dysfunction.^17^, ^18^ A potential downside of a dorsal technique is that the female urethral sphincter muscles are thicker dorsally (12 o'clock position), theoretically increasing the risk of incontinence after surgery with a dorsal approach^5^, ^9^. In our series, dorsal urethroplasty demonstrated excellent long‐term success, and based on the aforementioned advantages, it is now our preferred approach, offering the most versatility in treating strictures up to the proximal urethra, with no reports of de novo incontinence. We believe that the lower success rate observed in ventral approaches, particularly ventral inlay, can be attributed to the technical complexity of reaching the most proximal healthy urethral mucosa for graft fixation. However, successful outcomes have been reported in the literature of ventral approaches with the maintenance of continence, noting the importance of both the sphincter and pelvic muscles forming the basis for maintaining continence.^19^, ^20^ Interestingly, following failed ventral inlay surgery, most patients required additional procedures, primarily dilation and achieved asymptomatic status afterwards, with only one patient eventually requiring dorsal urethroplasty.

Traditionally, surgical approaches for female urethral reconstruction were categorised into either dorsal or ventral techniques. In this context, “dorsal” referred to the urethral wall near the corporal bodies of the clitoris at the 12 o'clock position, while “ventral” pertained to the wall near the vagina at the 6 o'clock position.^6^, ^7^ However, this nomenclature was derived from male urethral anatomy in the erect position, where the dorsal urethra aligns with the corporal bodies. Whilst this discrepancy and dorsal/ventral urethra is well described in the literature, it can cause confusion given the variation in anatomy between the male and female urethra.

Different criteria have been proposed to diagnose female urethral stricture in the literature. Khawaja et al. defined it as a flow rate of less than 10 ml/sec, the inability to calibrate the urethra at 10 Fr and the presence of proximal dilation observed on voiding cystourethrogram.^3^ Waterloos et al. and Onol et al., on the other hand, defined female stricture as the inability to calibrate at 14 Fr.^8^, ^11^ In this study, all patients who were included had a symptomatic presentation, a maximum urinary flow rate (Qmax) of less than 12 ml/sec. Furthermore, stricture disease was confirmed visually during cystoscopy and characteristics were further delineated with VCU.

Urethral dilatation is commonly employed as the initial therapeutic approach for managing female urethral stricture due to its perceived simplicity and familiarity within clinical practice.^4^ In a comparative study by Gülpinar et al., participants who ultimately underwent surgical interventions for female urethral stricture had typically undergone an average of two urethral dilatations before the surgical stage.^2^ Furthermore, the reported success rate of urethral dilatation stands at 41%, with a noticeable decline in success rates observed in cases involving multiple previous dilatations.^4^ Specifically, the success rate diminishes from 55.4% in women with no history of prior dilatation to a mere 14.9% in cases with a history of multiple prior dilatations.^4^ Additionally, it often necessitates subsequent dilations within an average timeframe of 15 months.^5^ In this study, 68% of patients had previously undergone urethral dilations. The mean number of dilatations administered before proceeding to urethroplasty was 9.0 underscoring the recurrent and fruitless nature of repeated dilatation therapy instead of definitive surgical intervention.

In our study, the mean age of presentation 45 years, corroborating the existing literature which has been reported in studies to range between 40 and 60 years.^1^ The primary aetiology underlying female urethral stricture in our study was idiopathic in nature (Table 1). This is consistent with existing literature which cites idiopathic and iatrogenic factors as the predominant underlying aetiology.^1^, ^2^ Idiopathic was defined in this cohort as a diagnosis of exclusion, in the absence of other cause including trauma, iatrogenic, prior radiotherapy, lichen sclerosis etc.

This study's limitations include a lack of sexual function evaluation post‐operatively. Further research on long‐term impact on sexual function would allow for a more comprehensive outcome comparison between urethroplasty techniques as they evolve. Furthermore, there is a need for a larger patient cohort to confirm the superiority of one surgical approach over the other, or to further identify patient or stricture factors that may have improved outcomes with a particular technique.

## 6 CONCLUSION

In conclusion, this multi‐centre study on the management of female urethral strictures underscores the pivotal role of urethroplasty as a highly efficacious and durable therapeutic option. Female urethral strictures, though infrequent, pose significant challenges in clinical practice, often necessitating long‐term solutions beyond traditional dilatations and intermittent catheterisation. With an overall success rate of 88%, it signifies the transformative potential of urethroplasty in enhancing the quality of life for female patients afflicted with urethral strictures as an enduring solution. The choice of surgical approach should be guided by consideration of stricture characteristics and location, further emphasising the necessity for personalised treatment strategies. While this study has provided substantial insights into the management of female urethral strictures, continued research and comparative analyses are imperative to refine surgical techniques and optimise patient care further.

## AUTHOR CONTRIBUTIONS

All authors contributed to writing, data collection and production. Concept generation by Devang Desai, Sanjay Kulkarni and Pankaj Joshi.

## CONFLICT OF INTEREST STATEMENT

The authors declare no conflicts of interest.
